# Hybrid genome assembly and phenotypic assays reveal carbohydrate metabolism diversity in *Lacticaseibacillus* strains

**DOI:** 10.1007/s00253-026-13940-9

**Published:** 2026-06-29

**Authors:** Emanuele Della Monica, Worarat Kruasuwan, Natnicha Wankaew, Tantip Arigul, Thidathip Wongsurawat, Camilla Lazzi, Monica Gatti, Alessia Levante

**Affiliations:** 1 Department of Food and Drug, University of Parma, 43124 Parma, Italy; 2 Oxford Nanopore Centre of Excellence (CoE), Research Department, Faculty of Medicine Siriraj Hospital, Mahidol University, Bangkok, Thailand

**Keywords:** *Lacticaseibacillus* spp., Phenotype microarray, Hybrid genome assembly, Comparative genomics, CAZyme gene clusters, Carbohydrate metabolism

## Abstract

**Abstract:**

Investigation of carbohydrate metabolism in lactic acid bacteria is essential for the rational selection of strains for fermentation processes, particularly in emerging applications involving non-conventional substrates or building of synthetic microbial consortia. However, establishing robust genotype–phenotype relationships remains challenging, as gene presence alone often fails to explain observed metabolic traits without considering the genomic context and regulatory architecture. In the present study, we combined hybrid genome assembly (Illumina and Oxford Nanopore) with high-throughput phenotype profiling (Biolog GENIII and PM2A) to investigate carbohydrate utilization in five *Lacticaseibacillus* strains. Phenotypic assays revealed clear intra- and inter-specific variability in substrate utilization. We therefore investigated whether such differences could be attributed to the organization and regulatory context of carbohydrate-associated loci, rather than to gene presence alone. Functional annotation based on COG and CAZyme databases revealed candidate genomic regions potentially involved in carbohydrate metabolism. Comparative analysis between predicted and experimentally observed substrate usage highlighted specific loci associated with carbohydrate utilization profile. The trehalose (*tre*) operon was conserved across all strains, while at least two distinct cellobiose-associated loci were detected in each genome. Despite the presence of these loci, *L. paracasei* strains were unable to metabolize cellobiose, a phenotype likely linked to the presence of a downstream TetR-type transcriptional repressor within the cellobiose (*cel*) operon. Additionally, a genomic region uniquely found in *L. rhamnosus* strains was associated with gentiobiose utilization, consistent with phenotypic observations. Overall, these findings highlight the importance of integrating phenotypic validation with complete genome context to support the identification of candidate structural and regulatory determinants of carbohydrate utilization in lactic acid bacteria.

**Key points:**

• *Phenotype microarrays reveal metabolic traits of interest in isolated strains.*

• *Regulatory context is key to understanding carbohydrate metabolism differences.*

• *Basis of subspecies-dependent cellobiose metabolism in L. paracasei is provided.*

**Supplementary Information:**

The online version contains supplementary material available at https://doi.org/10.1007/s00253-026-13940-9.

## Introduction

Lactic acid bacteria (LAB) are a group of gram-positive, non-sporulating microorganisms commonly present in fermented foods as well as in the gastrointestinal tracts of humans and animals. Recognized as *Qualified Presumption of Safety* (QPS) and/or *Generally Recognized as Safe* (GRAS), these bacteria have also drawn significant attention for their probiotic properties and associated health benefits. Consequently, a wide array of industrial applications has been explored and established over time, particularly within the dairy, bakery, meat, beverage, vegetable, and legume sectors.

Their utilization in the food industry primarily stems from their metabolic versatility but proves also useful for converting a broad range of substrates into value-added compounds. LAB can degrade complex macromolecules—such as polysaccharides, proteins, and other bioactive compounds—into simpler, more readily bioavailable molecules. Additionally, they exhibit several biosynthetic capabilities, producing essential vitamins, antioxidant compounds, and flavor-enhancing metabolites (Wang et al. 2021). During catabolism, they produce organic acids (e.g., lactic acid) that lower the pH, thereby enhancing food preservation and inhibiting the growth of pathogenic and spoilage microorganisms (Yang et al. 2024). Moreover, some LAB are known to synthesize bacteriocins, further enhancing their antimicrobial potential and overall contribution to food safety and quality (Chen et al. 2025), and others are known to synthesize exopolysaccharides (EPS) with various biological activities (Wu et al. 2023). This complex ensemble of metabolic reactions may occur at different stages during food fermentations, when LAB may be exposed to a series of stress conditions (Sionek et al. 2024) (i.e., thermal, pH, osmotic, salt, or starvation), which drive their adaptation and/or specialization toward specific metabolisms. Among LAB genera, the *Lacticaseibacillus* genus has drawn attention due to its ecological flexibility and functional diversity, which becomes evident upon comparison with updated genera of the former *Lactobacillus* genus (Zheng et al. 2020), revealing a certain degree of phenotypic and niche-associated variability within its species members (You et al. 2023). Indeed, the species included in the *Lacticaseibacillus* genus have been described as possessing a nomadic lifestyle, as they can be isolated from various environments (Duar et al. 2017). Previous studies have identified niche-specific adaptations, including gene loss or acquisition associated with the transition to dairy environments (Fontana et al. 2018; Levante et al. 2021; Stefanovic and McAuliffe 2018) as well as metabolic diversity enabling the transformation of plant-based substrates into volatile compounds (Ricci et al. 2018), antimicrobials (Ricci et al. 2021; Figuccia et al. 2025), and even peptides with biological activities (Fadlillah et al. 2025; Tonini et al. 2024; Undhad Trupti et al. 2021). However, the genetic determinants of this potential are still untapped, due to limited studies linking the genome content and the metabolic properties of the strains.

One reason for this can be found in the limited toolbox for phenotyping studies, even if alternatives exist, such as the phenotype microarray platform. This method has been applied to screen the metabolic properties of lactic acid bacteria, revealing a certain degree of functional diversity among the strains, even from the same isolation niche (Bettera et al. 2023).

Hybrid assembly strategies, combining both short- and long-read sequencing technologies, enable the reconstruction of more accurate genomic structures and a deeper understanding of the genomic context in which specific sequences are embedded (Hu et al. 2021). These sequencing strategies are instrumental in elucidating the ecological roles of microbial species during fermentation, uncovering metabolic interactions, and identifying key functional traits for quality improvements (Somerville et al. 2022; Srinivas et al. 2022; Walsh et al. 2020). Additionally, WGS data offers a framework for the rational design of both synthetic and engineered microbial consortia tailored to specific fermentation processes. By predicting each member’s phenotypic capabilities and modeling community-level interactions, WGS-guided strategies enable the systematic assembly of stable, efficient consortia optimized for targeted functional outcomes (Jing et al. 2024; Rafieenia et al. 2022; Xu and Yu 2021). However, genomic databases lack sufficient depth for purely in silico pathway reconstruction, making targeted phenotypic assays and broad omics validation essential. Therefore, integrating experimental and computational data refines metabolic models and allows the discovery of novel loci, thereby improving functional annotation.

In this study, we applied an integrated genomic and phenotypic approach to five *Lacticaseibacillus* strains to investigate carbohydrate utilization patterns. The selected strains belong to the closely related species *Lacticaseibacillus rhamnosus* and *Lacticaseibacillus paracasei*, but despite their phylogenetic proximity, they exhibit phenotypic variability in carbohydrate utilization. Among these substrates, we focused on disaccharides, particularly β-glucosides, such as cellobiose and gentiobiose, as representative substrates to explore whether differences in utilization could be explained by genomic context and regulatory features rather than by gene content alone. Genes related to carbohydrate utilization metabolism were investigated, while functional annotation was compared to phenotypic profiles measured using Biolog GENIII and PM2A microarrays. Using these data, we investigated whether phenotypic variability among the strains could be described. This led us to identify candidate loci linked to carbohydrate utilization and to formulate hypotheses regarding operon organization and expression. The observed intra- and inter-specific metabolic diversity, detected at both genomic and phenotypic levels, allowed us to identify candidate regions associated with the utilization of trehalose, cellobiose, and gentiobiose. This difference could be attributed to the regulatory architecture of the operons, which could support the lack of gentiobiose utilization in the analyzed *L. paracasei* strains. More broadly, this work aims to advance a more predictive understanding of carbohydrate metabolism in LAB. Such knowledge is essential for the targeted selection and application of strains with defined metabolic capabilities, supporting the development of tailored fermentation processes in both food and broader biotechnological applications.

## Materials and methods

### Bacterial strains and culture conditions

*Lacticaseibacillus* strains 1019, 1473, 2333, 4186, and 2306, hereafter abbreviated as Lr1019, Lr1473, Lr2333, Lp4186, and Lp2306, respectively, belonging to the University of Parma Culture Collection (UPCCO), were isolated from dairy matrices, and details about strain isolation are described elsewhere (Levante et al. 2021). Bacterial strains were maintained as frozen stocks (−80 °C) in Man Rogosa Sharpe (MRS) medium (Oxoid, Milan, Italy) supplemented with 15% (w/v) glycerol. The cultures were propagated three times using a 2% (v/v) inoculum in 6 mL of MRS in sterile glass tubes and incubated under anaerobic conditions (AnaeroGen, Oxoid, Milan, Italy) at 37 °C for 15 h to ensure active growth prior to experimental assays.

### Metabolic profiling of strains using PM and GENIII microarrays

Revitalized strains were cultured with a 2% (v/v) inoculum in 6 mL of MRS medium in sterile glass tubes and incubated for 9 h. Afterward, 1 mL of bacterial culture was washed three times with 1 mL of Ringer solution to prepare the inoculum. Chemically Defined Medium (CDM) for PM2A carbon source microarrays was prepared according to the manufacturer’s instructions (Biolog Inc., Hayward, USA), with 2 mM MgCl_2_, 1 mM CaCl_2_, 50 µM of L-glutamate, 12.5 µM L-cystine, 25 µM UMP-2Na, 25 µM L-arginine, 25 µM hypoxanthine, 5 µM β-NAD, 0.25 µM riboflavin, 0.005% yeast extract, and 0.005% Tween 80. The cells were then diluted in PM inoculating fluid, prepared with the previously mentioned CDM, to achieve 60% transmittance, as measured using a Biolog Turbidimeter, before inoculating the PM2A and GENIII microarrays (Biolog Inc., Hayward, USA). Metabolic activity for PM2A was assessed by incubating the inoculated microarrays at 37 °C for 42 h, measuring absorbance intensity at 590 nm every 10 min. GENIII microarrays were inoculated at a transmittance of 95% and measured at 590 nm at 0 and 72 h, followed by background correction. An absorbance threshold of 0.1 was applied to discriminate between positive and negative substrate utilization. Data from PM2A microarrays were background-corrected and subsequently analyzed in RStudio v4.5.1 using the Growthcurver R package v0.3.1 (Sprouffske and Wagner 2016), which enabled the calculation of the auc_e parameter. A threshold value of 2 was applied to classify substrate utilization. This parameter was used as a reference for constructing a heatmap with the ComplexHeatmap package v2.24.1, which also includes a hierarchical clustering method based on Euclidean distance (Gu et al. 2016). Principal component analysis (PCA) on auc_e values was performed with FactoMineR v2.12 and visualized with factoextra v1.0.7 R packages (Husson et al. 2006; Kassambara and Mundt 2016).

### Total genomic DNA isolation, library preparation, and sequencing

For short-read sequencing, the analysis was performed as reported in a previous study (Levante et al. 2021). Briefly, total genomic DNA (gDNA) was isolated from 1 mL of overnight cultures prepared as described above using the DNeasy Blood & Tissue Kit (Qiagen, Italy), following the manufacturer’s protocol for the lysis of gram-positive bacterial cells. A total of 1 µg of DNA from each sample was used to prepare the libraries with the Illumina Nextera XT Library Prep Kit (Illumina, USA). Library quality was assessed using the Agilent Bioanalyzer High Sensitivity DNA Kit (Agilent Technologies, Italy), and quantification was performed with the Qubit High Sensitivity DNA Kit (Thermo Fisher Scientific, Italy). Sequencing was carried out on the Illumina MiSeq platform (Illumina, Italy) using 2 × 150 bp paired-end reads.

For long-read sequencing, total DNA was extracted using the ZymoBIOMICS DNA Miniprep Kit (Zymo Research, USA) following the manufacturer’s protocol. DNA quantification, used to assess extraction yield and suitability for library preparation, was performed with the QuantiFluor® ONE dsDNA System (Promega, Italy). Library preparation was carried out using the Rapid Sequencing DNA V14 Barcoding Kit (SQK-RBK114.24; Oxford Nanopore Technologies, ONT, UK) with 200 ng of input DNA per sample. Sequencing was conducted on a MinION Mk1B device (ONT, UK) using an R10.4.1 flow cell (FLO-MIN114; ONT, UK).

### Data pre-processing and hybrid genome assembly

Illumina reads were quality checked using FastQC v0.11.9 (Andrews 2010), adapters were removed, and low-quality reads (*Q* ≤ 30) were filtered out using fastp v0.23.2 (Chen et al. 2018). To obtain the highest accuracy from ONT long-read sequences, Dorado v0.8.0 (Oxford Nanopore Technology 2026) was used for basecalling in super accuracy (SUP v5.0.0) mode. The quality and adapter trimming of raw sequenced reads obtained from ONT was performed by Porechop v0.2.4 (Wick 2018) and Filtlong v0.2.1 (Wick 2025) for filtering, keeping only reads over 1000 base pairs and with a *Q* ≥ 10. NanoPlot v1.38.0 was used to evaluate the resulting reads (De Coster et al. 2018). Long-read and short-read sequences are publicly available on NCBI (BioProject: PRJNA694562).

To construct the genome, a hybrid assembly of both long-read and short-read was performed using Unicycler v0.4.8 (Wick et al. 2017). Consequently, the assembled genome was then checked for completeness and contamination using CheckM v1.2.1 (lineage_wf) (Parks et al. 2015) and MOB-suite v3.0.5 (mob_recon) was used for plasmid typing (Robertson and Nash 2018). The genome features were evaluated by QUAST v5.0.2 (Gurevich et al. 2013), and plasmid contigs were verified by searching against the Plasmer database (Zhu et al. 2023). Subsequently, genome annotation was performed with Prokka v1.14.6 (Seemann 2014), and taxonomic classification of the assembled genome was conducted using Kraken2 v2.1.3 (Wood et al. 2019). Average nucleotide identity (ANI) of strains against NCBI reference genomes was estimated with FastANI v1.34 (Jain et al. 2018). Circular visualization of assembled genomes was constructed with Anvi’o v8 (Eren et al. 2020), and the built-in pyANI function was used to calculate the ANI across genomes.

### Microbial functional annotation

Functional gene annotation for the five constructed genomes was performed using clusters of orthologous genes (COG) and classified and analyzed with the COG database, with COGclassifier v1.0.5 (https://github.com/moshi4/COGclassifier) used for processing. CAZyme gene clusters (CGCs) and their associated substrate specificities were predicted using run_dbcan v4.1.4 (https://github.com/linnabrown/run_dbcan) in conjunction with the dbCAN3 database (Zheng et al. 2023). Briefly, run_dbcan is part of the dbCAN3 standalone pipeline, and it identifies carbohydrate-active enzyme (CAZyme) genes based on HMM, DIAMOND, and Hotpep annotations, and clusters them into CGCs by evaluating the proximity of functionally related genes within the genome. To compare the genomic and phenotypic similarities of the strains included in this study with reference strains, the same pipeline—Prokka, COGclassifier, and run_dbcan—was used to predict carbohydrate-associated gene clusters (CGCs) linked to specific substrate metabolisms. Reference genomes metadata were retrieved from BacDive, which also provided curated phenotypic information, enabling direct comparison of substrate utilization profiles, by selecting assemblies with the highest quality score when available.

## Results

### *Lacticaseibacillus* genome construction and quality assessments

**Fig. 1:**
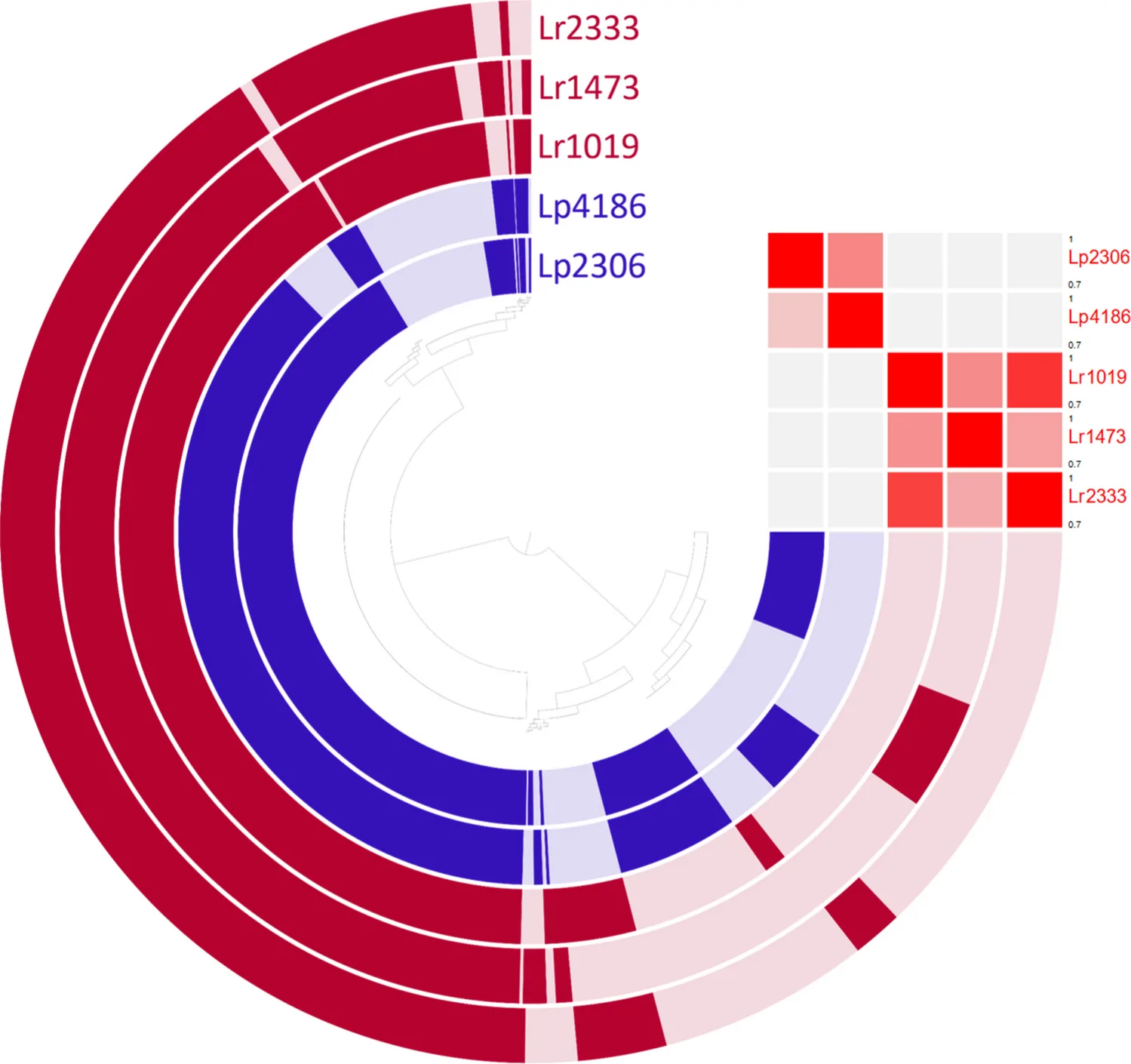
Circular representation of the genomes. *Lacticaseibacillus rhamnosus* strains (Lr1019, Lr1473, Lr2333) are highlighted in red, while *Lacticaseibacillus paracasei* strains (Lp2306, Lp4186) are highlighted in blue. Genome features are annotated in the legend. A heatmap displays the average nucleotide identity (ANI) percentage values among the analyzed strains, calculated by pyANI

Hybrid genome assemblies were generated using both Oxford Nanopore and Illumina sequencing platforms. Raw read statistics analysis is described in the Supplementary Table S1, and the principal genomic features of the resulting assemblies are summarized in Table 1. Taxonomic classification of the assembled genomes using Kraken2 revealed that strains 1019, 1473, and 2333 belonged to *Lacticaseibacillus rhamnosus* (Lr), with 94.04%, 90.12%, and 90.44% of reads assigned to the species, respectively. The remaining strains, 2306 and 4186, were classified as *Lacticaseibacillus paracasei* (Lp), with 97.60% and 99.20% of reads assigned to the species, respectively (Fig. 1 and Supplementary Fig. S1a). These two strains were further compared to NCBI reference genomes of *L.* *paracasei* subsp. *paracasei* (GCA_000155515.2) and *L.* *paracasei* subsp. *tolerans* (GCA_025946745.1) by FastANI. Both strains showed an ANI percentage closer to GCA_025946745.1 (98.21% for Lp2306 and 98.01% for Lp4186) than GCA_000155515.2 (98.20% and 97.91%, respectively). Genome completeness and contamination, as estimated by CheckM, exceeded 99% and remained below 0.2% across all assemblies (Table 1). Overall, genome sizes ranged from 2.77 to 3.08 Mbp. Among the Lr strains, genome sizes showed modest variation, from 3.00 Mb in strain Lr1019 to 2.98 Mb in Lr2333 and 2.94 Mb in Lr1473, with corresponding predicted coding sequences (CDSs) of 2793, 2781, and 2759, respectively (Supplementary Fig. S1b). Notably, plasmid content varied substantially across strains: Lr1473 harbored seven plasmids, whereas Lp2306 carried six, and Lr2333 contained only a single plasmid (Supplementary Figs. S1b and S2). Marked differences were also observed in the size and number of CDSs within each plasmid, with Lp2306 having the highest average plasmid size (approximately 30 kbp) and the greatest number of plasmid-encoded CDSs (208). No significant differences were observed in the number of annotated rRNA and tRNA genes across the strains (Table 1).

**Table 1:** Summary of hybrid-assembled genomic features of five *Lacticaseibacillus* strains

Strain	Molecule type	Size (bp)	GC (%)	CDS	rRNA	tRNA	Completeness (%)	Contamination (%)	Read length N50	Depth(×)
*Lacticaseibacillus rhamnosus* (Lr)										
Lr1019	Chromosome	3,004,620	46.7	2793	15	60	99.95	0.08	4869	253
	Plasmid 1	47,321	43.3	48	–	–	–	–		553
	Plasmid 2	13,457	42.4	13	–	–	–	–		3220
Lr1473	Chromosome	2,944,387	46.8	2759	15	61	100.00	0.1	6570	168
	Plasmid 1	34,026	43.9	42	–	–	–	–		329
	Plasmid 2	30,316	42.6	36	–	–	–	–		323
	Plasmid 3	17,813	45.1	17	–	–	–	–		1184
	Plasmid 4	8837	40.3	7	–	–	–	–		1153
	Plasmid 5	4073	39.6	4	–	–	–	–		865
	Plasmid 6	3390	43.1	5	–	–	–	–		1869
	Plasmid 7	1923	37.9	1	–	–	–	–		1304
Lr2333	Chromosome	2,987,373	46.8	2781	15	60	99.52	0.1	9537	116
	Plasmid 1	4939	43.7	5	–	–	–	–		900
*Lacticaseibacillus paracasei* (Lp)										
Lp2306	Chromosome	3,080,113	46.3	2920	15	59	99.00	0.19	8941	140
	Plasmid 1	8958	40.4	6	–	–	–	–		1148
	Plasmid 2	5996	46.6	7	–	–	–	–		1592
	Plasmid 3	44,313	43.4	52	–	–	–	–		154
	Plasmid 4	47,414	44.1	59	–	–	–	–		191
	Plasmid 5	5087	39.5	5	–	–	–	–		1475
	Plasmid 6	68,929	43.7	79	–	–	–	–		128
Lp4186	Chromosome	2,778,761	46.6	2670	15	60	99.61	0.0	10,434	109
	Plasmid 1	56,644	44.5	74	–	–	–	–		120
	Plasmid 2	41,658	42.5	50	–	–	–	–		114

### Metabolic profiles of *Lacticaseibacillus* strains

**Fig. 2:**
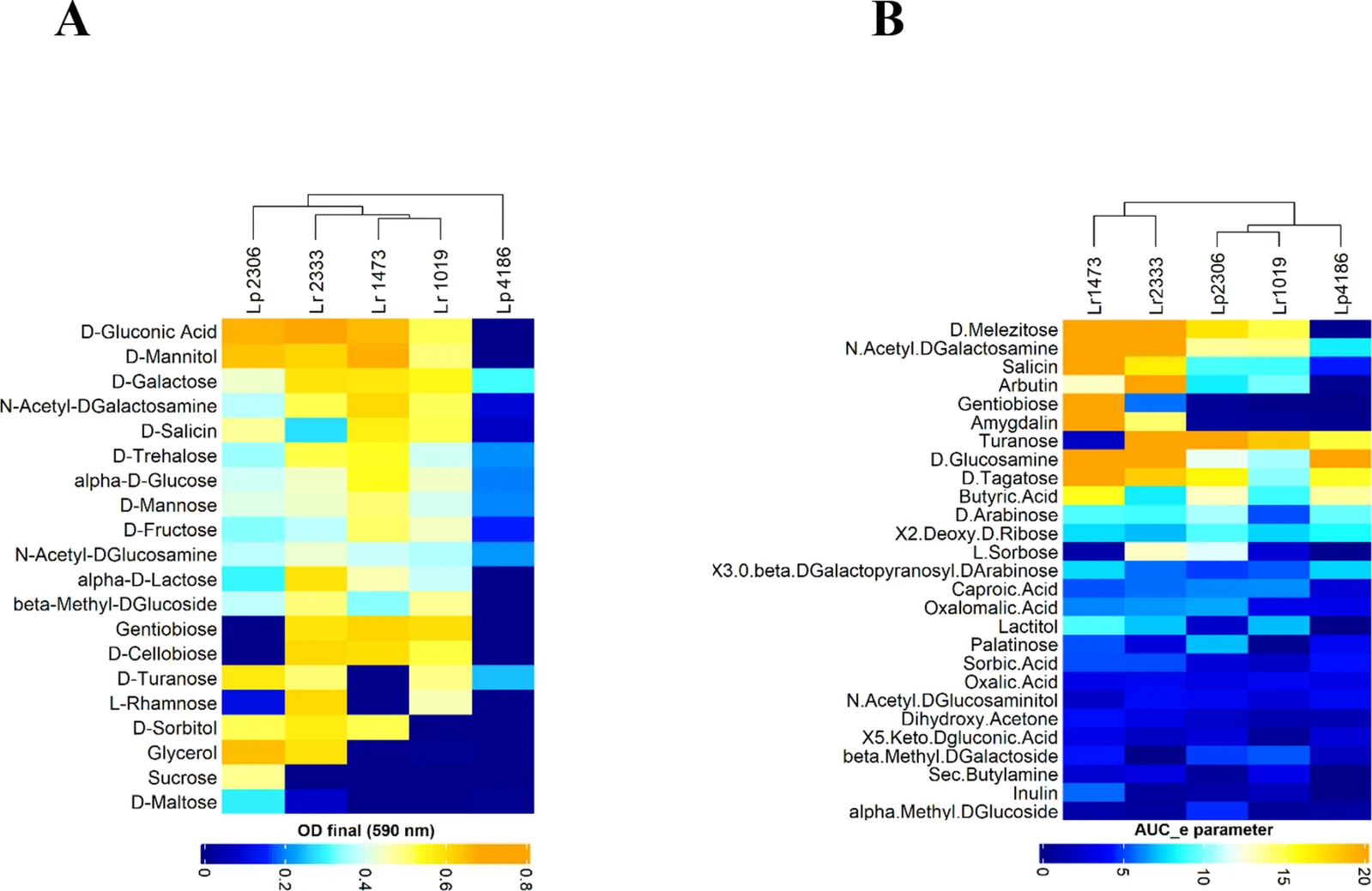
Heatmap representation of phenotypic profiles derived from GENIII (**A**) and PM2A (**B**) microarray assays. Color intensity reflects either the final optical density (OD590) measured 42 h post-inoculation or the area under the curve (AUCₑ) parameter, calculated using the growthcurver package in R

To identify genotype–phenotype associations, we first investigated the phenotypic differences across the five strains, using the observations to understand whether the differences could be linked to carbohydrate-associated genomic regions. To assess the metabolic profiles of the five *Lacticaseibacillus* strains, two phenotypic assays (GEN III and PM2A) were employed (Fig. 2 and Supplementary Fig. S3). The GEN III assay revealed species-specific patterns of carbohydrate utilization within 72 h post-inoculation. Hierarchical clustering based on Euclidean distance of GEN III metabolic profiles identified a well-defined cluster comprising strains Lr2333, Lr1473, and Lr1019, with Lp2306 showing greater similarity to this group than to the remaining isolate. By contrast, Lp4186 was clearly segregated from all other strains, exhibiting markedly lower overall metabolic activity and forming a distinct outgroup. Among the three strains (Lr2333, Lr1473, and Lr1019), no major metabolic differences were observed, except for the substrates D-turanose and L-rhamnose, which were not metabolized by strain Lr1473. However, some substrates such as gentiobiose and D-cellobiose appeared to be specifically utilized by this group (Fig. 2A).

Subsequent analysis using PM2A microarrays, specifically focused on carbohydrate utilization, further highlighted some inter- and intra-species metabolic diversities. Strains Lr1473 and Lr2333 displayed higher metabolic activity toward several di- and trisaccharides and glycosides compared with the other strains (Fig. 2B and Supplementary Fig. S3). Notably, none of the isolates exhibited substantial metabolic activity toward amino acids under the conditions tested. A core set of substrates, including D-tagatose, D-glucosamine, N-acetyl-D-galactosamine, salicin, and D-arabinose, was metabolized by all strains. Additional substrates were utilized by nearly all isolates, apart from strain Lp4186, which did not metabolize D-melezitose, arbutin, or lactitol, the latter displaying a low activity in the remaining strains, and strain Lr1473, which failed to metabolize turanose (Fig. 2B)*.* Strain Lp4186 was positioned closer to the cluster formed by Lr1019 and Lr1473 than Lp2306, indicating that it shares a higher degree of metabolic similarity with Lr strains for the substrates evaluated in the PM2A assay, despite being in a separate cluster in the PCA graph (Supplementary Fig. S4).

### COG comparative analysis of chromosomal and plasmid-encoded sequences

**Fig. 3:**
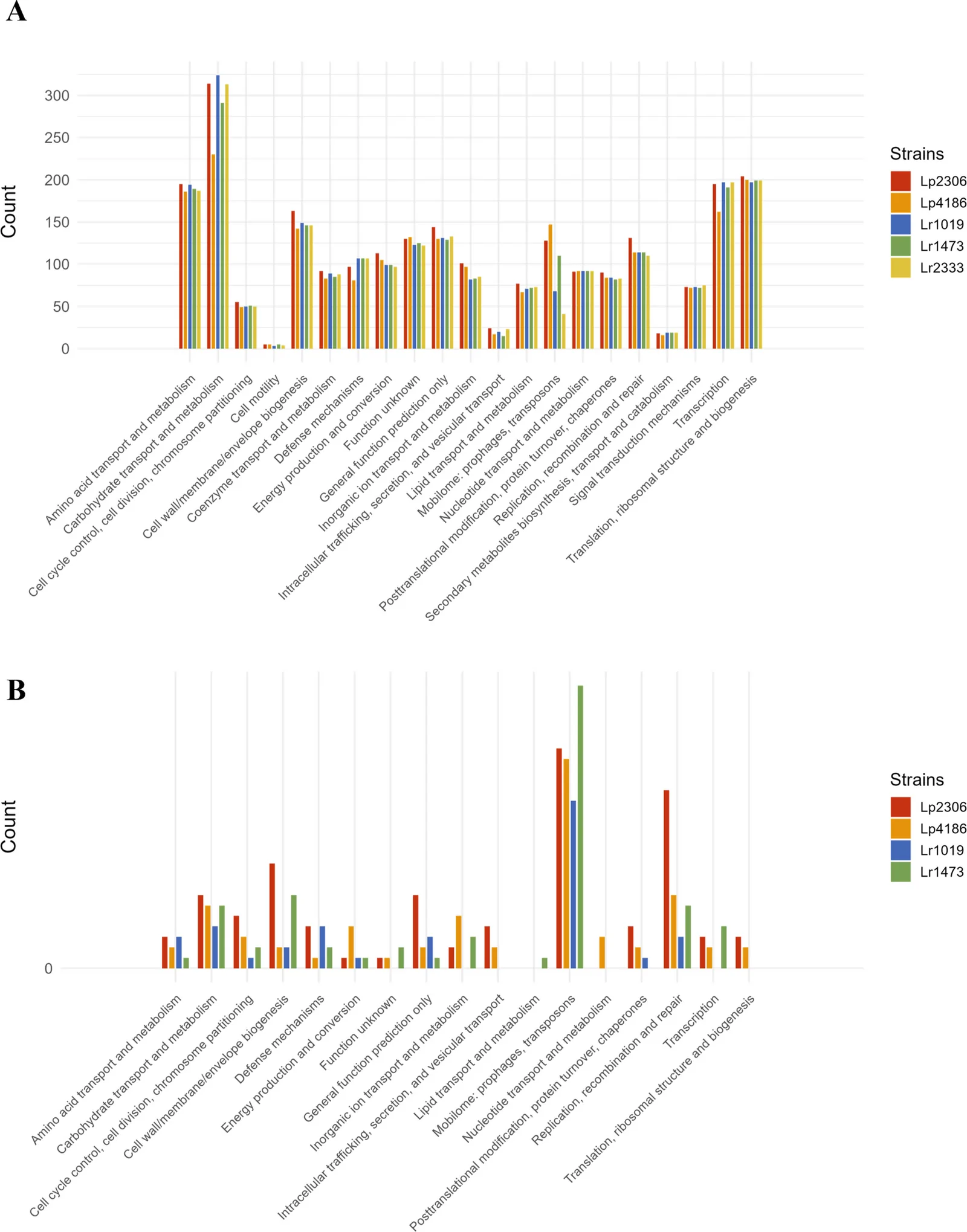
Grouped barplot of COG counts for genomes (**A**) and plasmids (**B**) by COGclassifier. COGs for each LAB (Lr1019, Lr1473, Lp2306, Lr2333, and Lp4186) were grouped into categories based on their annotated function; strain Lr2333 does not encode any plasmid-associated COGs and is therefore not included in the legend

To investigate the genetic basis underlying carbohydrate metabolism in the five *Lacticaseibacillus* strains, clusters of orthologous groups (COGs) were predicted across the five assembled genomes. Among *Lacticaseibacillus* strains, Lr1019 exhibited the highest number of annotated sequences in this category (324), followed by Lp2306 and Lr2333 (314 and 313, respectively). On the other hand, Lr1473 (291) and Lp4186 showed the lowest representation (230) in the chromosome (Fig. 3). “Carbohydrate transport and metabolism” was the most abundant functional category across bacterial chromosomes, followed by “transcription,” “translation, ribosomal structure and biogenesis,” and “amino acid transport and metabolism” categories. When the analysis was restricted to plasmid-encoded sequences, the most enriched categories were those associated with the mobilome and with replication, recombination, and repair. Lr1473 (7 plasmids) and Lp2306 (6 plasmids) were the most enriched strains in these respective categories. COG profiles for both genome- and plasmid-encoded sequences are shown in Fig. 3.

### Carbohydrate-active enzymes (CAZyme) annotation

**Fig. 4:**
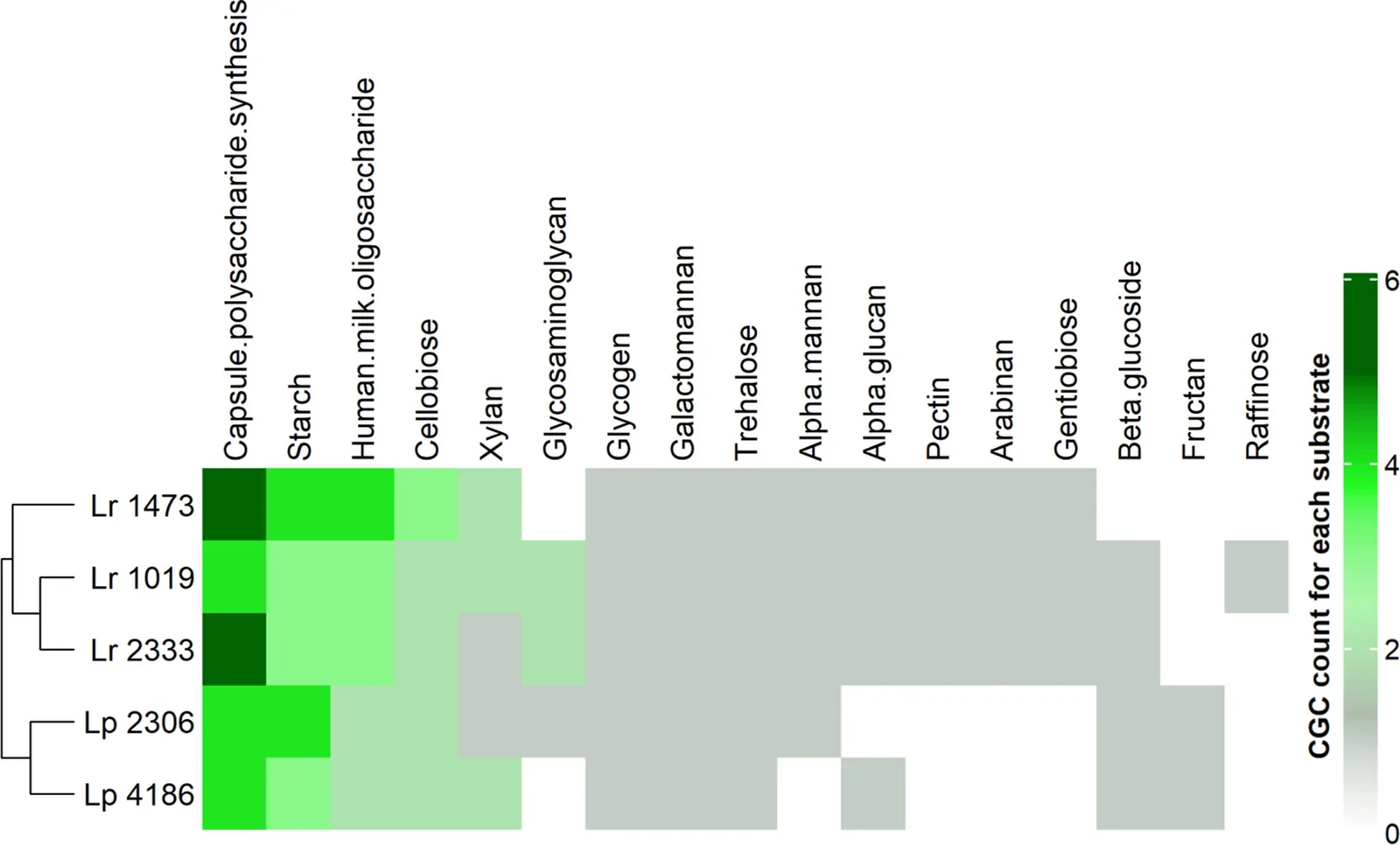
Heatmap showing the CGCs distribution associated with predicted substrates based on run_dbcan tool analysis across strains. Color intensity reflects the count of CGCs associated with the same substrate

The distribution of domains identified through the carbohydrate-active enzymes (CAZyme) database is shown (Fig. S5). Overall, no substantial differences were observed in either the type or abundance of CAZy domains across the analyzed strains. The most prevalent domains were GT4 and GT2, which encode glycosyltransferases, along with GH1 and GH13, which catalyze the hydrolysis of β-linked (primarily β−1,4) and α-linked (primarily α−1,4 and α−1,6) glycosidic bonds, respectively. Additional relevant domains included GH25, whose sole activity reported in CAZy is peptidoglycan endo-β−1,4-N-acetylmuramidase. The dbCAN3 standalone pipeline was subsequently used to identify CAZyme gene clusters (CGCs) and to predict substrate utilization across strains. Among the predicted substrates, capsule polysaccharide synthesis, starch, and human milk oligosaccharides were associated with the highest numbers of CGCs, with minor variation among strains (Fig. 4). Predicted metabolic capacities appeared broadly conserved at the intraspecies level. Notably, fructan utilization was absent from all three Lr genomes but present in the Lp genomes. Moreover, glycosaminoglycan, β-glucoside, and raffinose utilization were not predicted for Lr1473, in contrast to the other Lr strains. In Lp genomes, pectin, arabinan, gentiobiose, and raffinose utilization were not predicted relative to the Lr genomes. Additionally, α-mannan and glycosaminoglycan utilization were predicted exclusively in Lp2306, whereas α-glucan utilization was detected only in Lp4186 (Fig. 4).

### Comparing observed metabolic activities with predicted substrates

**Fig. 5:**
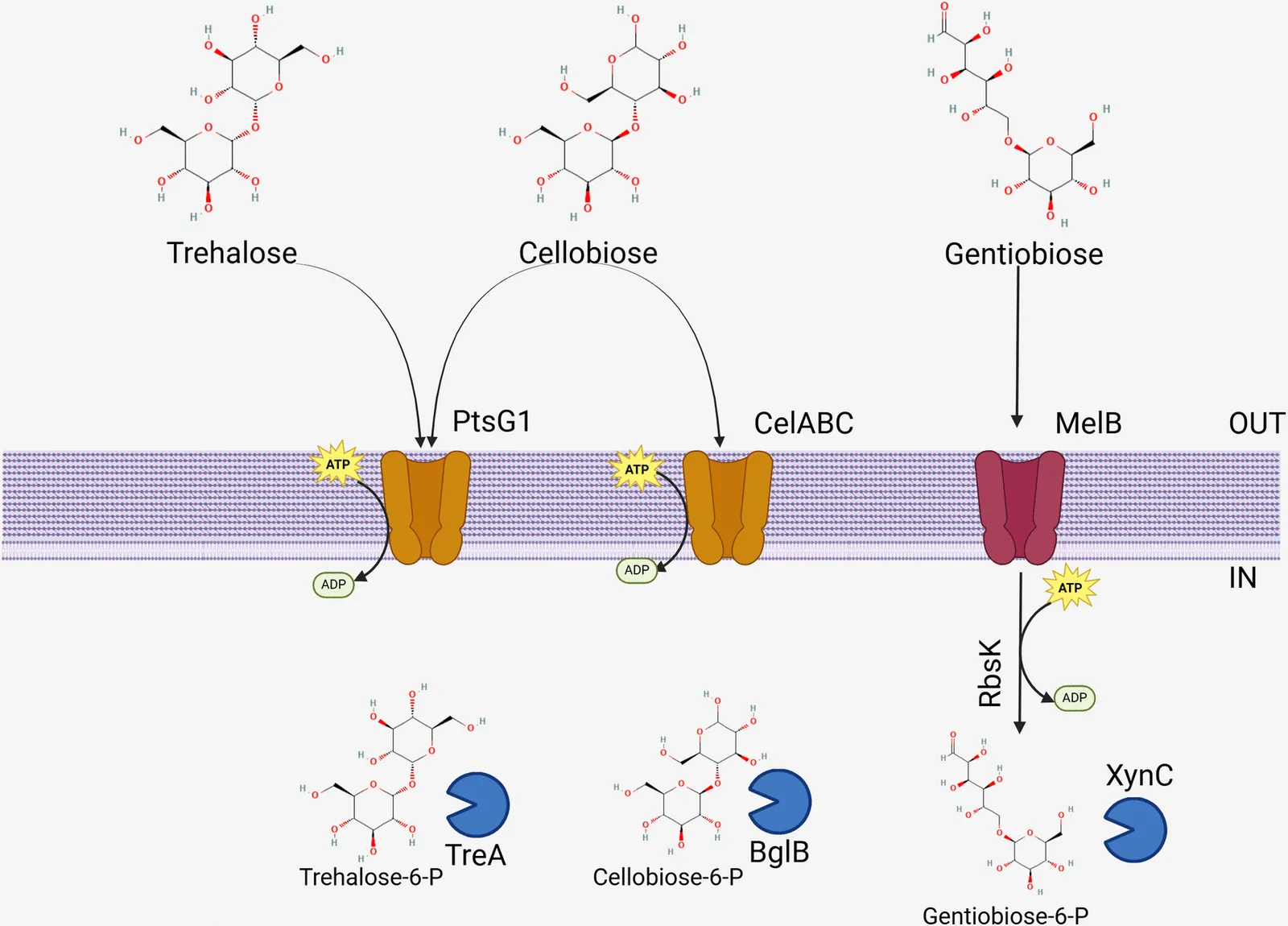
Schematic representation of the utilization pathways for trehalose, cellobiose, and gentiobiose in LAB. Trehalose is imported via PtsG1 and hydrolyzed by the glycoside hydrolase TreA. Cellobiose is taken up through either the CelABC transporter or PtsG1 and subsequently cleaved intracellularly by BglB. Gentiobiose is transported by the MelB symporter, phosphorylated by RbsK, and hydrolyzed by XynC. The resulting glucose units are then funneled into central LAB metabolism. Created with BioRender.com

**Fig. 6:**
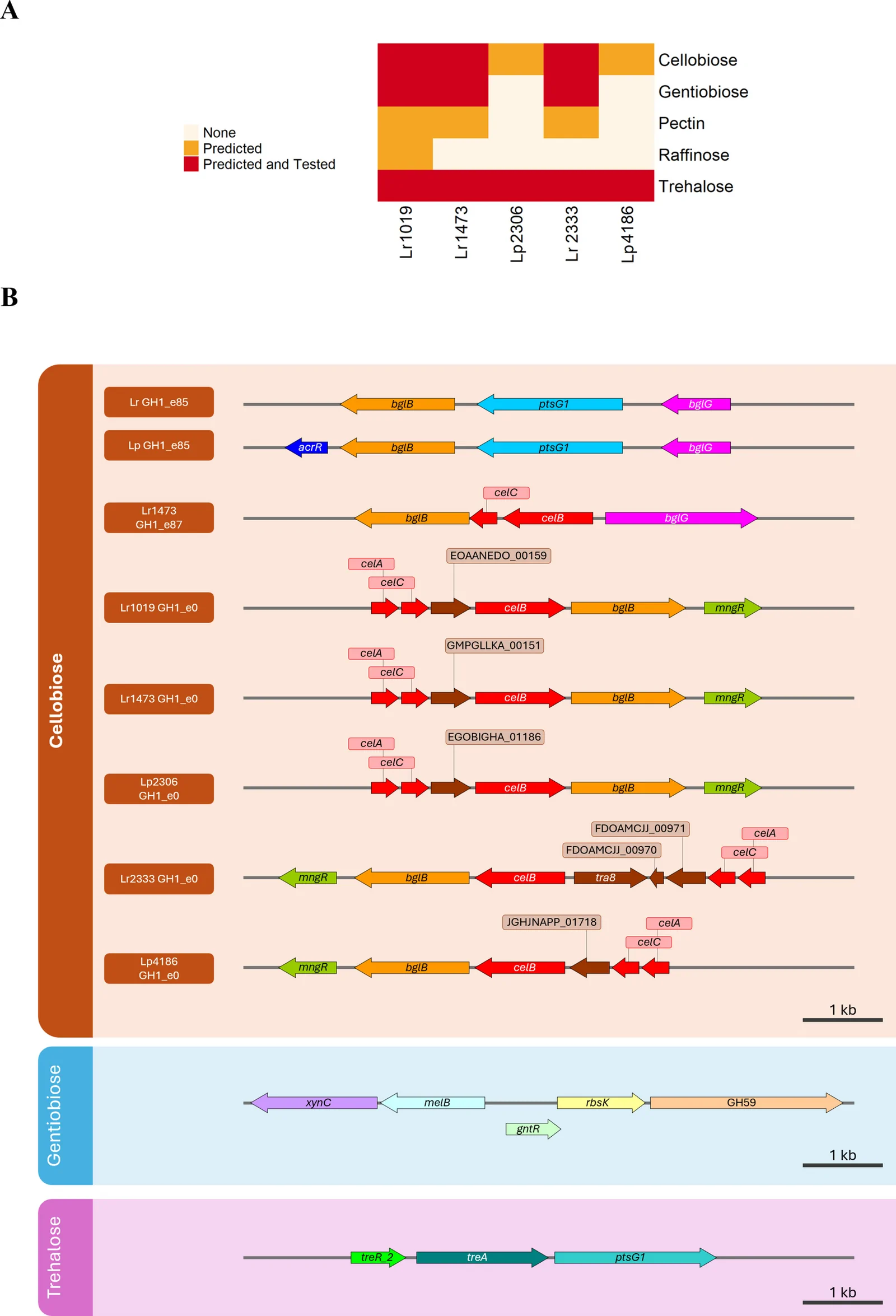
Heatmap illustrating the overlap between predicted substrate utilization and metabolic activity, as assessed using GENIII and PM2A phenotype microarrays (**A**). Schematic representation of genomic regions identified through CGC analysis and associated with the utilization of cellobiose, gentiobiose, and trehalose (**B**). For gentiobiose and trehalose, only the conserved CGC architecture shared among all Lr strains and between Lr and Lp, respectively, is shown. In contrast, all CGCs associated with cellobiose utilization are displayed for each strain, differentiated based on the glycoside hydrolase domain GH1 (GH1_e0, GH1_e85, GH1_e87)

Among the substrates predicted by the dbCAN3 standalone pipeline, only gentiobiose, cellobiose, trehalose, pectin, and raffinose overlapped with those tested in the phenotypic microarray assays. Consequently, the analysis focused on these compounds to assess the concordance between computational predictions and the corresponding metabolic phenotypes. To contextualize this comparison, Fig. 5 provides a schematic overview of the known pathways involved in the utilization of trehalose, cellobiose, and gentiobiose. The heatmap showed a good concordance for some substrates, particularly for trehalose, used by all Lp and Lr strains, and gentiobiose, utilized by all the Lr strains encoding for the necessary enzymes (Fig. 6). Interestingly, cellobiose was predicted to be metabolized by all strains; however, no metabolic activity was detected in the two Lp strains. Apparent inconsistencies were also noted for pectins, which were not metabolized by Lr strains despite showing predictive support, and for raffinose, whose utilization was predicted for Lr1019 but was not corroborated by the phenotypic assay (Fig. 6). Results for gentiobiose and cellobiose were further compared with metabolic profiles and substrate predictions performed over assemblies of reference strains of *L. rhamnosus* (BacDive ID 6427, GenBank ID AZCQ00000000), *L. paracasei* subsp. *paracasei* (BacDive ID 6548, GenBank ID AP012541), and *L. paracasei* subsp. *tolerans* (BacDive ID 6434, GenBank ID AYYJ00000000) retrieved by the BacDive database.

The CGCs associated with each substrate were examined in detail, leveraging the improved genomic context provided by hybrid assembly (Table 2). For trehalose, all strains possessed CGCs containing the GH13_e1 domain (GH13_29 in Lp4186) and a sequence annotated as *gntR*. Moreover, COG annotation revealed the presence of *amyA* (COG0366), a trehalose-6-phosphate hydrolase, also annotated as *treA*, flanked upstream by *mngR* (COG2188), a transcriptional repressor, also annotated as *treR_2*, and downstream by *ptsG1* (COG1263), a component of a phosphotransferase system (PTS).

**Table 2:** Summary of CGCs clusters distributed across genomes, each associated with a specific substrate predicted by run_dbcan tool

Carbon source	*L. rhamnosus*			*L. paracasei*	
	Lr1019	Lr1473	Lr2333	Lp2306	Lp4186
Cellobiose	GH1_e85	GH1_e85	GH1_e85	TetR_N GH1_e85	TetR_N GH1_e85
		GH1_e87			
	GH1_e0	GH1_e0	GH1_e0	GH1_e0	GH1_e0
Gentiobiose	GH30_9 *GntR* *pfkB* GH59	GH30_9 *GntR* *pfkB* GH59	GH30_9 *GntR* *pfkB* GH59	–	–
Pectin	GH78_e8/ CBM67_e28	GH78_e8/ CBM67_e28	GH78_e8/ CBM67_e28	–	–
Raffinose	GT8_e141 GT8_e165 GH36_e0	–	–	–	–
Trehalose	*GntR* GH13_e1	*GntR* GH13_e1	*GntR* GH13_e1	*GntR* GH13_e1	*GntR* GH13_29

Similarly, all strains harbored at least two CGCs linked to cellobiose utilization, each containing glycosidases annotated as GH1_e85 and GH1_e0 (Table 2). Lr1473 also featured a third CGC containing a GH1_e87 domain. Notably, a TetR-type transcriptional regulator was solely identified within the GH1_e85-containing CGCs of Lp2306 and Lp4186, while it was absent in all Lr strains. The genomic context of GH1_e0 (annotated as *bglB*, COG2723) was highly conserved, with *celA* (COG1440), *celB* (COG1455), and *celC* (COG1447) consistently upstream, and *mngR* downstream. Additional unannotated sequences were found among *celC* and *celB* sequences in all strains, except for Lp2306, which instead carried a *tra8* sequence (COG2826). In contrast, GH1_e85-containing CGCs, also classified as *bglB*, were followed by *ptsG1* and *bglG* (COG3711), a transcriptional antiterminator. In Lp strains, *acrR* (COG1309), a DNA-binding repressor, was also present upstream of *bglB*. Genomic context of GH1_e87 in Lr1473 resembled that of GH1_e0, but *bglG* was present instead of *mngR*, and lacked the *celA* sequence. Among the BacDive reference strains, strain 6427 possessed three CGCs containing an operon for cellobiose metabolism: two with an Lr GH1_e85-like structure and one with a GH1_e0-like structure. Strain 6548 also carried three CGCs, resembling Lr GH1_e85, Lp GH1_e85 (including the *acrR* sequence), and GH1_e0 structures. In contrast, strain 6434 possessed only two cellobiose-associated CGCs, one GH1_e0-like and one Lr GH1_e85-like; however, the latter is located at the beginning of a contig on the negative strand, preventing assessment of whether additional downstream sequences are missing. The NCBI reference genome GCA_025946745.1 showed a CGC distribution similar to strain 6548, although no phenotypic information on cellobiose metabolism was available for this strain.

Gentiobiose was the only substrate for which a complete overlap was observed between the presence or absence of metabolic activity and the presence or absence of specific CGCs. In all three Lr strains, the CGC associated with gentiobiose utilization contained a sequence encoding the GH30_9 domain, along with three additional sequences annotated as *gntR*, *pfkB*, and GH59. According to CAZyme, GH30 includes enzymes with various activities, notably β−1,6-glucosidase activity on gentiobiose, while GH59 is exclusively associated with ceramide β-galactosidase activity. COG annotation identified these sequences as *xynC* (COG5520), a glycoside hydrolase involved in cell wall biogenesis; *gntR* (COG1802), a DNA-binding transcriptional regulator; and *rbsK* (COG0524), a ribokinase involved in carbohydrate metabolism. GH59 did not yield a COG annotation. Additionally, a permease annotated as *melB* (COG2211)—a symporter of α-galactosides such as melibiose and raffinose—was located between *xynC* and *gntR* within the same CGC. Graphical representation of regions associated with the utilization of trehalose, cellobiose, and gentiobiose, along with a binary heatmap that shows overlapping among substrate predictions and phenotypic tests, is shown in Fig. 6. Interestingly, no gentiobiose-associated CGCs were detected in BacDive assemblies of reference strains, and metabolic profiles showed mixed results for gentiobiose degradation in *L. rhamnosus* reference strains, while being consistently negative in *L. paracasei* reference strains.

For pectin utilization, all three Lr strains encoded a CGC associated with a sequence annotated with dual domains GH78_e8/CBM67_e28, corresponding to α-L-rhamnosidase activity and a carbohydrate-binding module. COG annotation links this sequence to GDB1 (COG3408), a glycogen debranching enzyme with α−1,6-glucosidase activity. Remarkably, raffinose utilization is predicted only in Lr1019, which harbors a CGC containing two glycosyltransferase domains (GT8_e141 and GT8_e165) and one glycoside hydrolase domain (GH36_e0), associated with exo-α−1,6-galactosidase activity. The GT8 domains belong to two sequences both annotated as *rfaJ* (COG1442), while GH36_e0 corresponds to *galA* (COG3345), an α-galactosidase. These genes are separated by *plsC* (COG0204), an acyltransferase involved in phospholipid biosynthesis.

## Discussion

This study shows how differences in carbohydrate utilization among closely related *Lacticaseibacillus* strains may depend on operon organization and regulatory context, thus requiring a deeper level of investigation than gene presence alone. By integrating hybrid genome assemblies with phenotype profiling, we refined the interpretation of substrate utilization phenotypes beyond simple gene presence patterns, identifying candidate genotype–phenotype associations and proposing mechanistic hypotheses related to operon organization and regulatory architecture. Our findings should therefore be interpreted at three complementary levels: (i) describing phenotypic variability among strains, (ii) candidate genotype–phenotype associations supported by comparative genomics, and (iii) mechanistic hypotheses related to operon organization and transcriptional regulation, which remain to be experimentally validated.

The implementation of long-read sequencing technologies, along with short-read sequencing, enables the reconstruction of highly accurate genomic architectures, enhancing the reliability of functional annotations. Strain 2333 and strain 2306 were previously classified as *L. paracasei* and *L. casei*, respectively, based solely on a short-read assembly (Levante et al. 2021). However, integrating both short- and long-read sequencing allowed the construction of a complete genome and improved the accuracy of species identification, ultimately reclassifying these strains as *L. rhamnosus* and *L. paracasei*, respectively. When hybrid assemblies are integrated with high-throughput phenotype profiling, this approach provides a powerful framework for genotype–phenotype matching. Such comprehensive analyses facilitate the identification of gene-level variations that underpin metabolic diversity across strains (Bayjanov et al. 2013; Buron-Moles et al. 2019; Ceapa et al. 2015). From the perspective of this study, this approach not only broadens our understanding of the five investigated strains, but also allows the identification of putative regulatory mechanisms for selected phenotypes, by the integration of complete genome sequences and phenotype profiling. In this study, metabolic diversity was investigated across five strains, three assigned to the species *L. rhamnosus* (Lr) and two to *L. paracasei* (Lp). These five isolates were chosen because they are among the most extensively characterized *Lacticaseibacillus* strains within our UPCCO collection and had already been employed in previous studies by our group, for their capability to adapt to various substrates outside their isolation niche (Ricci et al. 2018, 2021; Saadoun et al. 2022), thus providing a well-defined background for comparative analysis. Although limited in size, the selected strain set provides a controlled comparative framework for hypothesis generation regarding phenotype–genotype matching. This hypothesis can be supported by comparison with other data from collections such as BacDive and subsequently tested on larger collections of strains. COG annotation revealed interspecies differences in carbohydrate transport and metabolism, consistent with the divergent substrate utilization profiles revealed by GENIII and PM2A microarrays, while still allowing the identification of a core subset of substrates that highlights a shared specialization towards specific compounds. Notably, Lp4186 exhibited the narrowest metabolic repertoire, whereas the three Lr strains showed broad, largely overlapping activities with minor intraspecific variation. When examining substrate categories, Lp2306 and Lr1019 appeared to preferentially utilize monosaccharides and glycosides, suggesting strain-specific specialization in metabolic capabilities towards certain substrate categories. Interestingly, the substrate utilization patterns obtained from GENIII and PM2A microarrays were broadly consistent with those reported for other *Lacticaseibacillus* strains in the BacDive database (Schober et al. 2025). For cellobiose, *Lacticaseibacillus paracasei* subsp. *paracasei* (BacDive ID 6548) is described as positive in API 50CH assays, whereas subsp. *tolerans* (BacDive ID 6434) is reported as negative, in agreement with the Lp2306 and Lp4186 strains. Similarly, the phenotype of the Lr strains aligned with that of the corresponding reference strain available in BacDive (BacDive ID 6427). These strains were selected as external reference, since they are among the most widely represented in culture collections. A comparable qualitative concordance was also observed for raffinose and trehalose utilization. For gentiobiose, subsp. *tolerans* is consistently reported as negative, matching our results, whereas subsp. *paracasei* and *L. rhamnosus* display more heterogeneous records in BacDive. COG categories related to amino acid transport and metabolism, as well as transcription, translation, ribosomal structure, and biogenesis, were abundant across all strains, reflecting the nomadic lifestyle of this specific genus of LAB and their ability to adapt to diverse environments and nutrient availability (Duar et al. 2017; Zheng et al. 2020). Metabolic functionality assessment might prove insightful, alongside large-scale pangenomic studies, to determine whether genomic diversity translates into differential functionality for certain substrates, such as carbohydrates (You et al. 2023). Plasmids were mainly associated with the mobilome (prophages and transposons), though categories linked to nutrient utilization were also present, suggesting a role in adaptive mechanisms. Additionally, encoding toxin-antitoxin systems, these plasmids are likely to be stably inherited across generations by the strains harboring them (Folli et al. 2017; Levante et al. 2021).

To examine the concordance between predicted and observed metabolic patterns, genomic regions associated with substrate utilization were investigated. Trehalose-related sequences were conserved across all strains and corresponded to a canonical *tre* operon containing a periplasmic trehalase, a transcriptional regulator, and a PTS transporter (Shrestha et al. 2024), consistent with the phenotypic results. This feature may be particularly relevant for innovative fermentations, such as those involving red algae (Rhodophyta), where trehalose has been detected alongside minor polyols (Hagemann and Pade 2015) or insects, in which it represents the main circulating sugar in the hemolymph (Tellis et al. 2023).

Conserved gene clusters associated with gentiobiose metabolism have been identified across all Lr strains capable of metabolizing this substrate. These CGCs contained *xynC*, *melB*, *gntR*, *rbsK*, and a gene encoding a GH59 domain-containing protein. Although *xynC* encodes a glycoside hydrolase domain from GH30 family, typically linked to the degradation of β-linked disaccharides, its specific role in gentiobiose catabolism remains unresolved. Nonetheless, its consistent presence exclusively in gentiobiose-positive Lr strains, and its absence in Lp, suggests a functionally relevant role. It is unclear whether *xynC* operates within a complete operon under the transcriptional control of *gntR*, whether its enzymatic activity is selective for gentiobiose, or whether it participates in a broader carbohydrate metabolic network. However, this correlation supports the hypothesis that *xynC* may serve as a key enzymatic component in gentiobiose utilization, potentially acting as the primary driver of its catabolism in Lr. It is well established that gentiobiose degradation requires the coordinated activity of a gentiobiase and a gentiobiose phosphorylase (Palmer and Anderson 1972). The consistent genomic proximity of *rbsK*, encoding a ribokinase, to *xynC* further supports the existence of a dedicated metabolic pathway, possibly involving phosphorylation-dependent cleavage mechanisms. Importantly, no gentiobiose-associated CGCs were detected in the BacDive 6427 assembly, despite mixed phenotypes reported for this strain, suggesting the presence of an alternative genomic region involved in gentiobiose metabolism. Gentiobiose is also a constituent of amygdalin. Notably, amygdalin utilization was consistent across all Lr strains, whereas it was absent in Lp strains. This trait may therefore be advantageous in the fermentation of ripe fruits and seeds, which are characterized by the presence of this uncommon sugar, potentially reducing the bitterness associated with amygdalin (Zhang et al. 2024).

Regarding cellobiose utilization, two CGCs characterized by the glycoside hydrolase domains GH1_e0 and GH1_e85 were conserved across all strains, with Lr1473 carrying an additional GH1_e87-containing region that exhibited an intermediate architecture compared with the other two, and, like them, followed the canonical organization of the *cel* operon (Parker and Hall 1990), although lacking the *celA* component of the transporter. The GH1_e0 and GH1_e85 regions both contained the canonical celABC transporter, which is typically associated with cellobiose uptake. Instead, the GH1_e85 region included *ptsG1*, a transporter classified as a PTS IIC component specific for glucose, maltose, and N-acetylglucosamine. While the previous results suggest a potential link between this genomic region and cellobiose utilization, the functional divergence from the other CGCs may reflect broader substrate specificity that influences operon activity beyond cellobiose metabolism.

All GH1_e0 CGCs shared the presence of an unclassified sequence located between *celC* and *celB*. In Lp2306, this region included *tra8*, an IS30 family transposase. A downstream *mngR* transcriptional regulator was also consistently present (Fig. 5). This architecture suggests the requirement for inducer molecules not present under assay conditions or potential regulatory interference by IS30 elements (Dalrymple 1987; Olasz et al. 2022).

In CGCs containing GH1_e85, the *cel* operon maintains its canonical structure across all Lr strains, with the sole variation being the presence of a single gene encoding *ptsG1*, a glucose-, maltose-, and N-acetylglucosamine-specific transporter. In Lp strains, however, a downstream sequence annotated as *acrR* was identified, which is associated with TetR family transcriptional repressors (Cuthbertson and Nodwell 2013). This suggests that *acrR* may represent a candidate regulatory element controlling *cel* operon expression in Lp, although this hypothesis requires experimental validation. Notably, *acrR* represents the primary structural difference between the operons of the two species. A similar organization has also been observed in *Escherichia* *coli* K12, with the characterization of the *celD* sequence, encoding a transcriptional repressor (Parker and Hall 1990). It is well established that *L. rhamnosus* can utilize cellobiose. In contrast, cellobiose metabolism in *L.* *paracasei* appears to be subspecies-dependent, as previously described: *L. paracasei* subsp. *paracasei* can metabolize this disaccharide, whereas *L. paracasei* subsp. *tolerans* lacks this capability. The underlying cause of this phenotypic divergence remains unclear and may be attributed either to the absence of the *cel* operon or to differences in regulatory mechanisms. In the present study, the Lp strains analyzed exhibit average nucleotide identity (ANI) values closer to *L. paracasei* subsp*. tolerans* than *L. paracasei* subsp. *paracasei*, while consistently lacking cellobiose utilization activity. Comparison with reference strains available in public databases further highlighted the importance of complete genome reconstruction for genotype–phenotype interpretation. The BacDive 6427 assembly displayed cellobiose-associated genomic regions consistent with those identified in our Lr isolates. In BacDive 6548 assembly, the presence of an Lr GH1_e85-like structure aligned with the observed activity toward cellobiose. In contrast, for BacDive 6434 assembly, the GH1_e85-like operon was located at the beginning of a contig, preventing assessment of whether an *acrR* sequence was present. Moreover, the NCBI reference genome lacked phenotypic information on cellobiose metabolism. These observations show a limitation of fragmented reference genomes: although gene content may appear conserved, incomplete assemblies can obscure operon architecture and prevent evaluation of regulatory hypotheses. Altogether, these observations suggest that strain-dependent traits, such as cellobiose degradation, may be driven by specific genomic configurations that become fully interpretable only with complete assemblies.

The combined evidence from cellobiose and gentiobiose metabolism illustrates how hybrid-assembly-supported genomic context refines the interpretation of phenotype beyond simple gene presence or absence. For instance, although Lr strains encoded GH78_e8 and CBM67_e28 domains, they showed no activity toward pectin, highlighting that the presence of individual CAZyme domains is insufficient to support the degradation of structurally complex polysaccharides. A similar pattern emerged for raffinose: Lp2306 metabolized galactose and sucrose but not raffinose, consistent with the absence of an α-galactosidase gene, whereas Lr1019 encoded a putative α-galactosidase yet did not metabolize raffinose, likely reflecting limited substrate specificity. These cases underscore how enzymatic function, regulatory context, and operon completeness collectively shape carbohydrate utilization phenotypes.

## Conclusions

This study demonstrates that carbohydrate utilization phenotypes in *Lacticaseibacillus* cannot always be predicted solely by gene presence and may depend on operon organization and regulatory context. Combining the phenotypic and genomic profiles not only confirms functional assignments but also provides a baseline for deeper investigation into gene organization and regulation. Integration of long- and short-read sequencing markedly improves genome reconstruction, reducing assembly fragmentation and thereby enhancing the accuracy of functional annotation. This improvement is particularly critical for tools that rely on genomic context, which perform optimally on complete rather than highly fragmented assemblies. Applied to a selected comparative set of *Lacticaseibacillus* strains, this approach enabled genotype–phenotype matching and the identification of candidate gene-level differences underlying carbohydrate metabolism diversity. Across the investigated substrates, gentiobiose utilization showed a strong association with the presence of a strain-specific genomic region, whereas cellobiose metabolism highlighted the importance of regulatory architecture in shaping metabolic phenotypes. While the results presented here remain putative and require further experimental validation, the framework effectively narrows down the genomic search space, facilitating targeted comparative analyses of regions potentially associated with phenotypic variation and contributing to a more predictive understanding of LAB metabolism to support the selection of strains for biotechnological applications.

## Supplementary Information

Below is the link to the electronic supplementary material.

ESM 1 (PDF 618 KB)

## Data Availability

Cabon source utilization data for PM2A microplates are available on Zenodo ([https://doi.org/10.5281/zenodo.19227448], (https:/doi.org/https://doi.org/10.5281/zenodo.19227448)). Genome sequences are available on NCBI under accession number PRJNA694562.
