# Supplementary material for: Hybrid genome assembly and phenotypic assays reveal carbohydrate metabolism diversity in Lacticaseibacillus strains
Source: Appl Microbiol Biotechnol. 2026 Jun 29;110:275. doi: 10.1007/s00253-026-13940-9 (PMC13578153; doi:10.1007/s00253-026-13940-9)
Supplement: Supplementary file 1 [file 253_2026_13940_MOESM1_ESM.pdf]

Supplementary material

Hybrid genome assembly and phenotypic assays reveal carbohydrate metabolism diversity in *Lacticaseibacillus* strains

Emanuele Della Monica[a], Worarat Kruasuwan[b]*, Natnicha Wankaew[b], Tantip Arigul[b], Thidathip Wongsurawat[b], Camilla Lazzi[a], Monica Gatti[a], Alessia Levante[a]**

Supplementary Figures

A

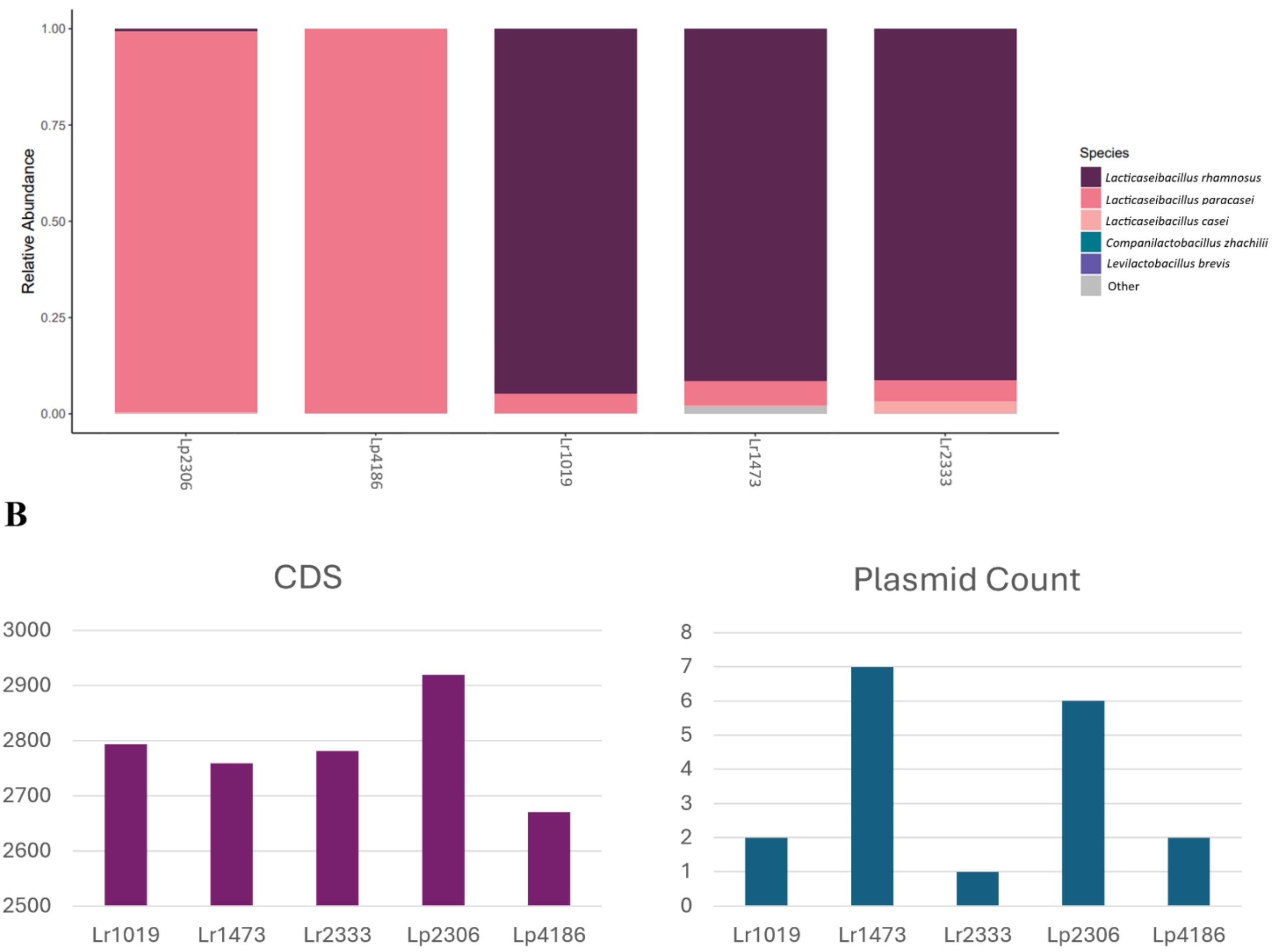

Supplementary Fig. S1. (A) Stacked barplot showing the taxonomic classification results obtained with Kraken2 for each strain, up to species level. (B) Left barplot showing the number of coding sequences (CDSs) annotated by Prokka from hybrid assemblies for each strain. Right barplot showing the number of plasmids identified by MOB-suite from the same hybrid assemblies.

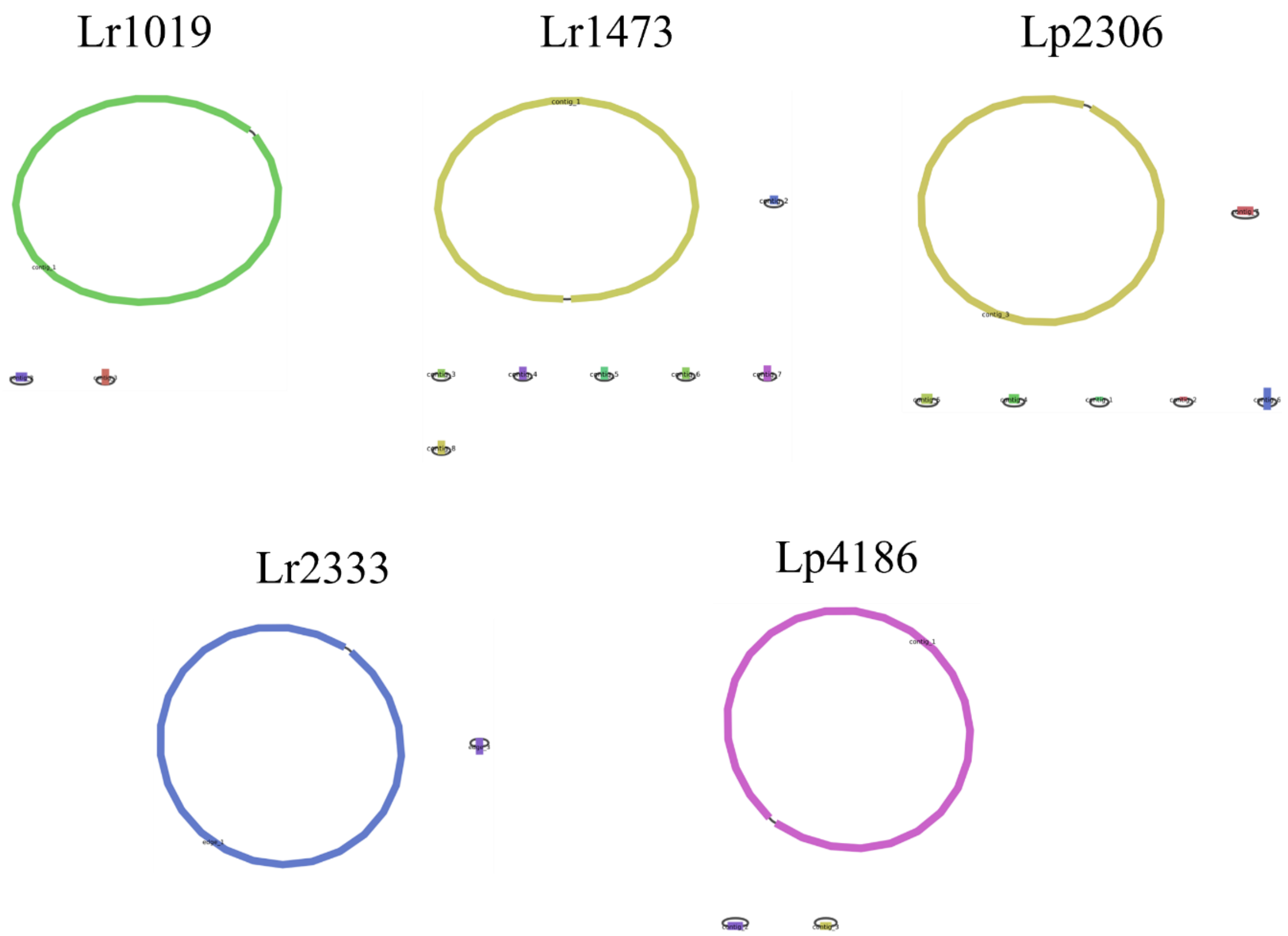

Supplementary Fig. S2. Bandage images showing the contigs obtained from hybrid genome assembly for each strain.

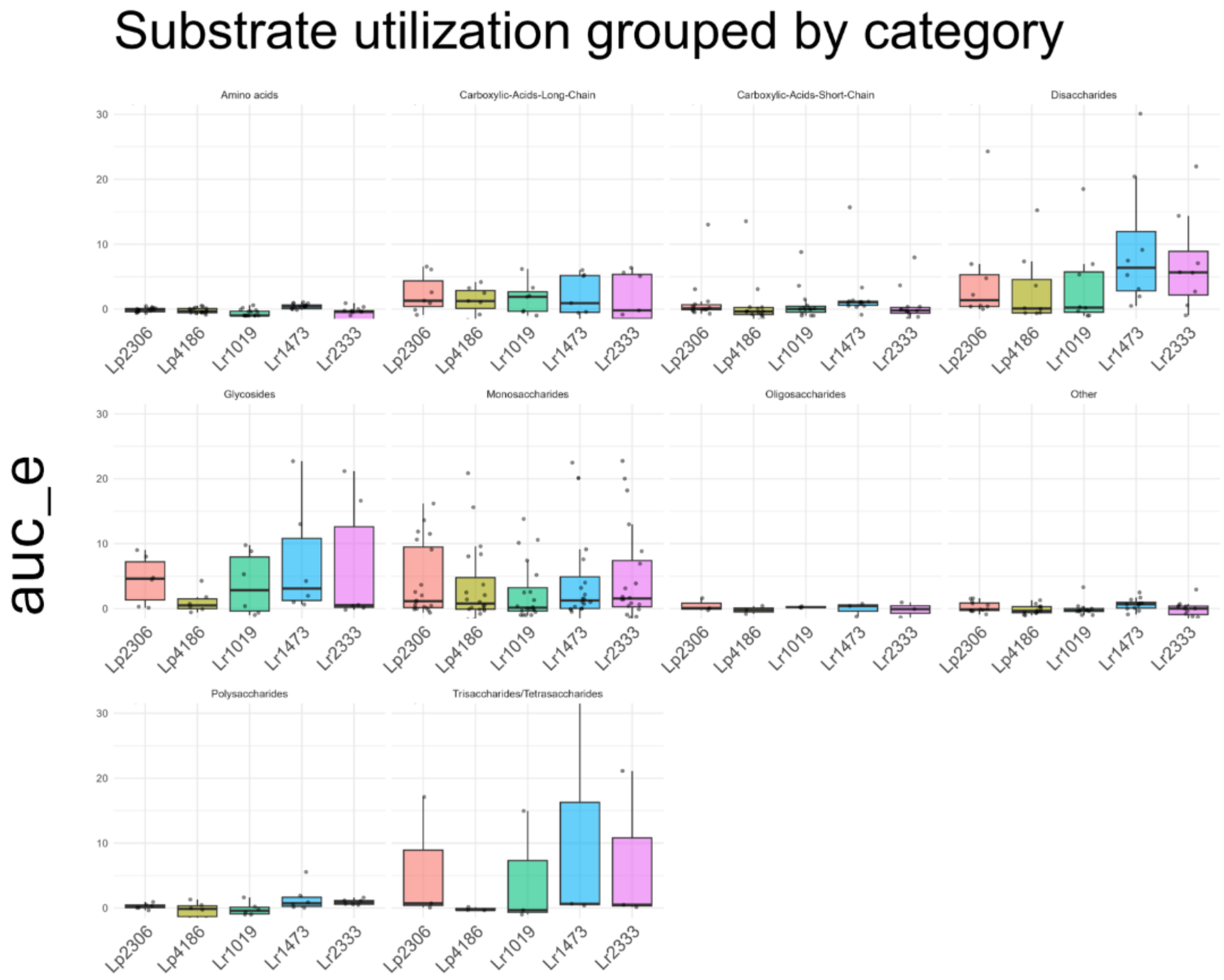

Supplementary Fig. S3. Boxplot per substrate category for PM2A phenotypic assays, showing the distribution of activity across strains. The activity is defined by the auc_e values calculated by the growthcurver R package.

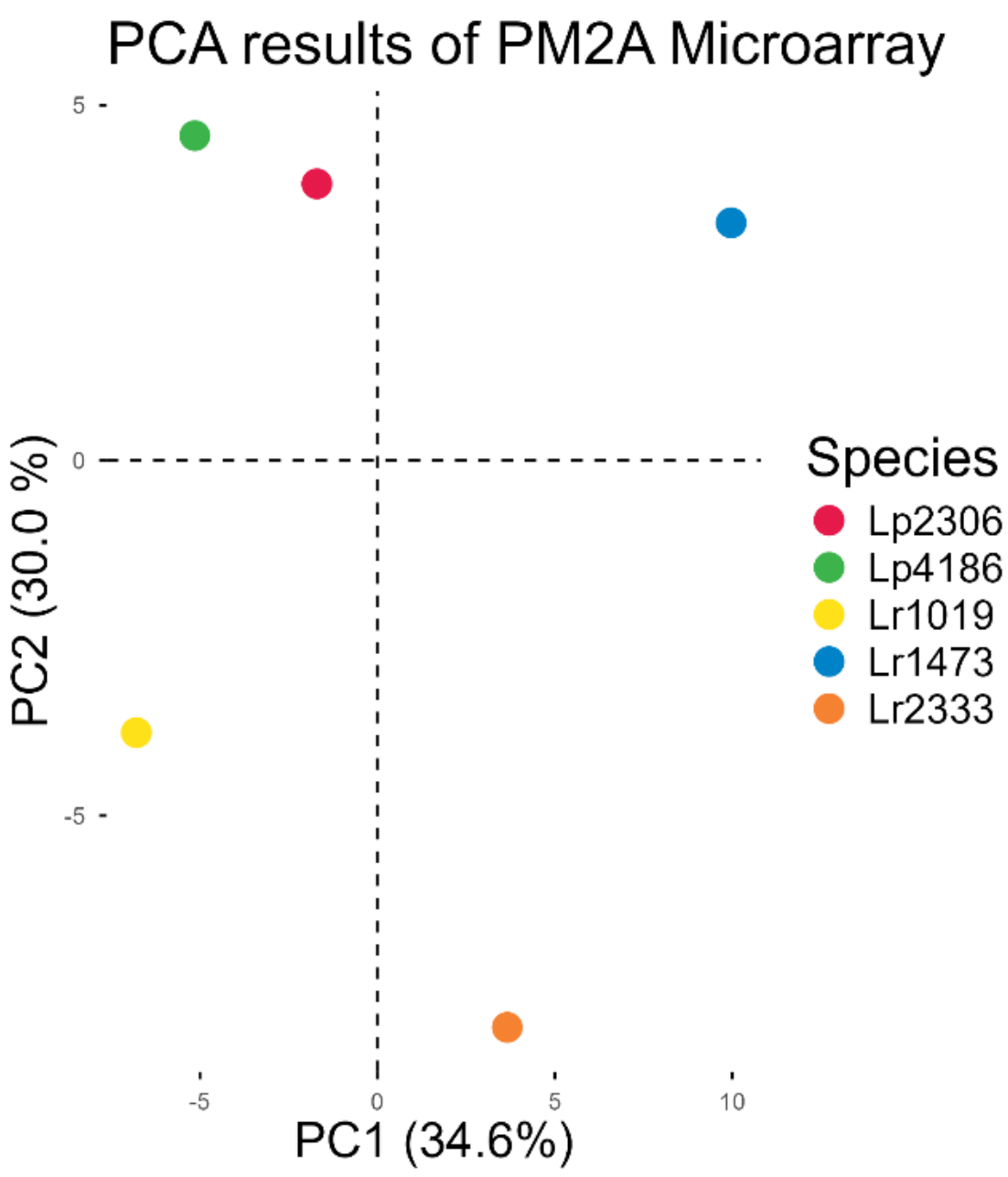

Supplementary Fig. S4. PCA graph based on auc_e values obtained using the *growthcurver* package from PM2A microarray data

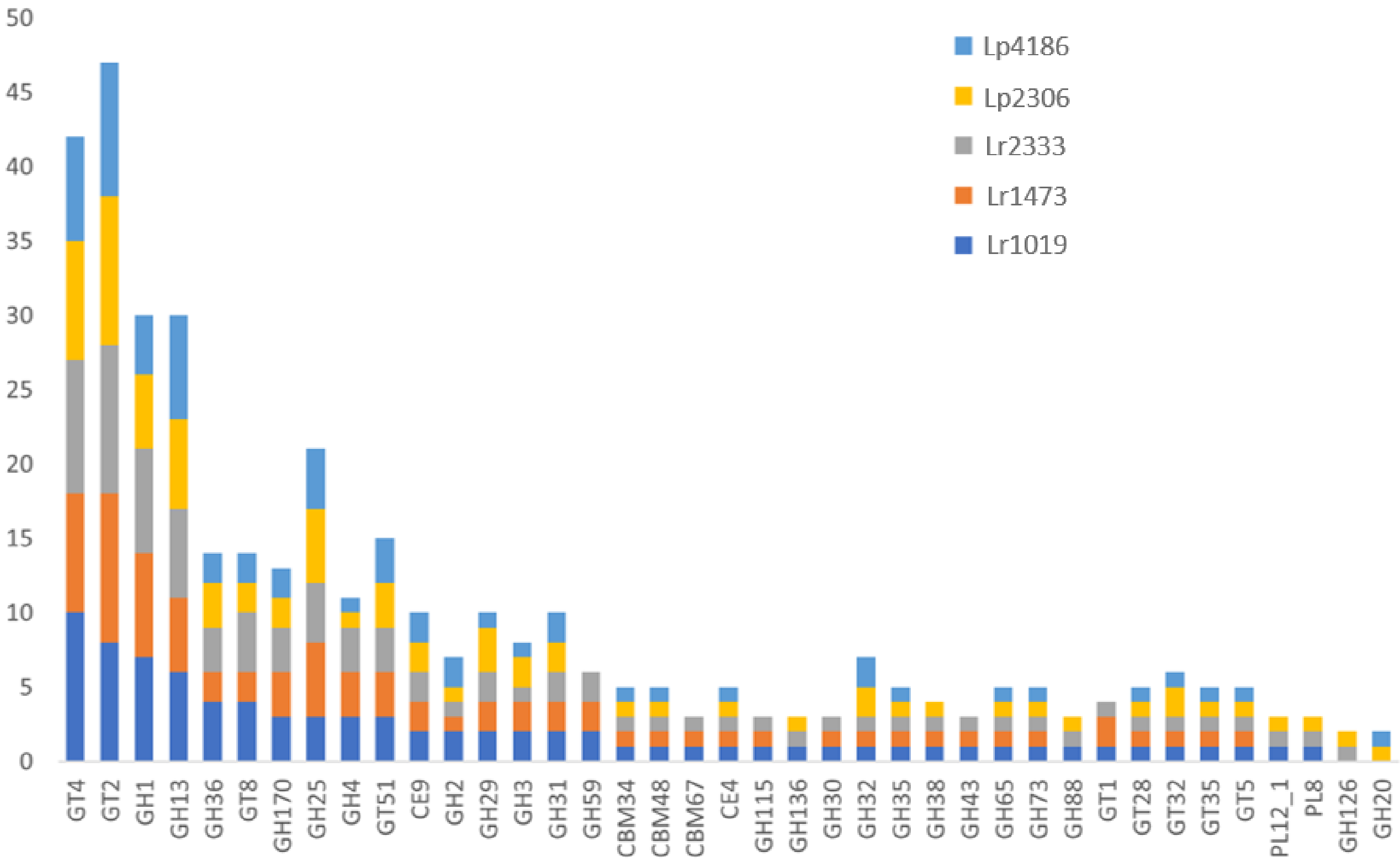

Supplementary Fig. S5. Stacked barplot showing the total count of genes assigned to each CAZy domain across all analyzed strains, as identified by the run_dbcan pipeline.

Supplementary Tables

Supplementary Table S1. Raw sequences of five strains obtained from both Nanopore long-read and Illumina short-read sequencing.

Strain ID	Total bases (bp)	No. of reads	Mean read quality (Q)	Mean read length (bp)	Read length N50
Nanopore long-read sequences					
Lr1019	1,035,392,990	435,963	22.8	2,375.0	4,869
Lr1473	1,523,912,340	532,005	22.8	2,864.5	6,570
Lr2333	663,520,178	143,825	23.0	4,613.4	9,537
Lp2306	632,997,922	157,239	22.7	4,025.7	8,941
Lp4186	462,257,751	107,374	23.0	4,305.1	10,434
Illumina short-read sequences					
Lr1019	832,293,364	3,011,950	>30	138	-
Lr1473	556,146,746	1,939,794	>30	143	-
Lr2333	540,461,138	1,860,190	>30	145	-
Lp2306	509,383,415	1,762,088	>30	144	-
Lp4186	313,568,915	1,084,934	>30	144	-
